# Fatal Spontaneous Rebleeding of Subdural Haematoma Following COVID-19 Infection: A Case Report

**DOI:** 10.7759/cureus.98623

**Published:** 2025-12-07

**Authors:** Rimsha Ghufran, Sana Aleem, Winluck Shayo, Abdul Hannan Siddiqui

**Affiliations:** 1 Internal Medicine, University Hospitals of Derby and Burton NHS Foundation Trust, Derby, GBR; 2 Acute Medicine, University Hospitals of Derby and Burton NHS Foundation Trust, Derby, GBR; 3 Cardiology, University Hospitals of Derby and Burton NHS Foundation Trust, Derby, GBR

**Keywords:** cerebrovascular accidents, covid-19, intracranial haemorrhage, spontaneous rebleeding, subdural hematoma

## Abstract

COVID-19 is associated with various neurological complications, including intracranial haemorrhage. However, spontaneous rebleeding of pre-existing subdural haematomas triggered by COVID-19 infection remains exceptionally rare. We report an 88-year-old woman with rheumatoid arthritis who presented with acute-on-chronic subdural haematoma following a fall. Conservative management was pursued, given her advanced age and comorbidities. After seven days of clinical stability, she contracted COVID-19 via PCR screening. Within 24 hours, she experienced catastrophic neurological deterioration (Glasgow Coma Scale declining from 15 to 3) without trauma or anticoagulation. Imaging confirmed worsening subdural haemorrhage with increased midline shift (12 mm) and acute rebleeding. Despite supportive care, she died 10 days later. This case demonstrates a temporal association between COVID-19 infection and fatal subdural haematoma rebleeding, possibly mediated through vascular injury, neuroinflammation, and coagulopathy. Patients with pre-existing intracranial haemorrhage who develop COVID-19 may require heightened neurological surveillance.

## Introduction

The COVID-19 pandemic has revealed the profound neurotropism and vasculopathic potential of SARS-CoV-2, with intracranial haemorrhage representing one of the most devastating neurological complications [1]. Cerebrovascular events (both ischemic and haemorrhagic) are the most common neuroradiologic abnormality among COVID-19 patients (27%) [2]. While de novo intracranial haemorrhage in COVID-19 has been increasingly documented, spontaneous rebleeding of pre-existing subdural haematomas triggered by SARS-CoV-2 infection, particularly without trauma or anticoagulation, remains exceptionally rare in the literature [3,4].

Subdural haematomas typically result from traumatic tearing of bridging veins, with elderly patients at the highest risk due to brain atrophy and vascular fragility [5]. Although recurrence rates following initial presentation range from 5-30% and are traditionally associated with coagulopathy, repeated trauma, or surgical manipulation [6], the interaction between COVID-19 and pre-existing subdural collections remains poorly understood. We present a case of an elderly woman who experienced fatal spontaneous subdural haematoma rebleeding within 24 hours of COVID-19 diagnosis, occurring without trauma or anticoagulation. This case illustrates the potential for COVID-19 to precipitate catastrophic haemorrhagic complications in patients with pre-existing intracranial pathology.

## Case presentation

An 88-year-old woman presented to the emergency department following a backwards fall with head trauma that occurred two days before presentation. She reported initially feeling well enough to remain at home, but subsequently developed progressive confusion and unsteadiness, prompting medical evaluation. Her medical history was significant for rheumatoid arthritis on methotrexate and etanercept, and hypertension.

On initial examination, the patient appeared mildly confused (Glasgow Coma Scale 14-15) but hemodynamically stable, with vital signs within normal limits. Initial laboratory investigations revealed significant pancytopenia (platelets 75×10⁹/L, haemoglobin 68 g/L, and white blood cell count 1.47×10^9^/L), attributed to her chronic methotrexate therapy. Coagulation parameters were within normal limits (prothrombin time 9.8 seconds, international normalized ratio, IRN, 0.87, activated partial thromboplastin time, aPTT, 18.6 seconds, fibrinogen 4.19 g/L).

**Figure 1:**
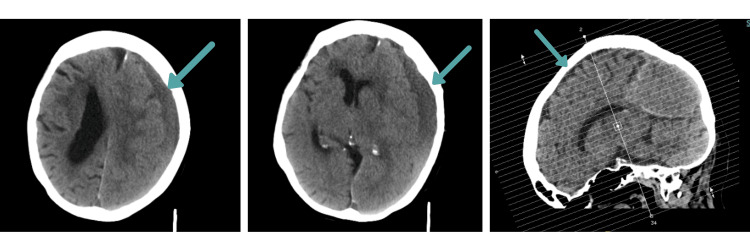
Initial CT head showing acute-on-chronic left subdural haematoma Non-contrast axial CT demonstrating left cerebral convexity subdural haematoma measuring 16 mm with posterior hyperdense layering, 9 mm midline shift, subfalcine and uncal herniation, and secondary hydrocephalus.

Non-contrast computed tomography (CT) of the head revealed an acute-on-chronic left cerebral convexity subdural haematoma with posterior hyperdense layering measuring up to 16 mm (Figure 1). The haemorrhage demonstrated significant mass effect with partial effacement of the left lateral ventricle and midline shift measuring 9 mm. Associated findings included left subfalcine herniation, left uncal herniation, effacement of the third ventricle, and dilatation of the right temporal horn, suggestive of developing obstructive hydrocephalus. No skull fracture was identified.

Given the patient's advanced age, comorbidities, and frailty, neurosurgical consultation recommended conservative management rather than surgical evacuation. Her immunosuppressive medications were discontinued, and she was monitored with serial neurological examinations.

**Figure 2:**
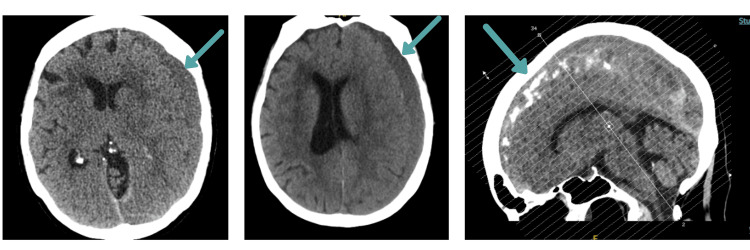
Follow-up CT head after inpatient fall CT demonstrating continued evolution of chronic subdural haematoma without acute rebleeding. Mass effect and midline shift remained stable compared to admission imaging.

Two days following admission, the patient sustained an unwitnessed inpatient fall. Repeat CT imaging performed per protocol demonstrated continued evolution of the left subdural haematoma; importantly, there was no measurable acute rebleeding component, and mass effect remained stable. Despite this mechanical trauma, no new haemorrhage occurred (Figure 2). Conservative management was continued.

Over the subsequent five days, the patient demonstrated gradual clinical improvement with resolution of confusion. She was mobilising with assistance, and plans were being formulated for discharge to a rehabilitation facility. Serial full blood count monitoring showed persistent pancytopenia with gradual improvement in platelet count (Table 1).

**Table 1:** Serial full blood count during admission *Day 7/8, following COVID-19 diagnosis. WBC - white blood cell count

Full blood count	Admission	Day 4	Day 6	Post-COVID-19*	Reference
Haemoglobin (g/L)	68	75	84	85	120-150
WBC (×10⁹/L)	1.47	2.24	2.25	1.15	4.0-11.0
Platelets (×10⁹/L)	75	36	41	84	150-400
Neutrophils (×10⁹/L)	0.7	1.29	1.31	0.51	2.0-7.5
Lymphocytes (×10⁹/L)	0.62	0.61	0.67	0.36	1.0-4.0

On the seventh day of admission, routine PCR screening detected SARS-CoV-2 infection. The patient was asymptomatic with stable respiratory status and no supplemental oxygen requirements; COVID-19 was classified as mild. No COVID-19 specific therapies, including antivirals, corticosteroids, or anticoagulation, were administered, given her stable respiratory status and significant haemorrhagic risk from pre-existing subdural haematoma.

Within 24 hours of COVID-19 diagnosis, without any intervening trauma or change in anticoagulation status, the patient experienced precipitous neurological deterioration. She became increasingly drowsy and had a rapid decline in consciousness (Glasgow Coma Scale deteriorating from 15 to 3).

**Figure 3:**
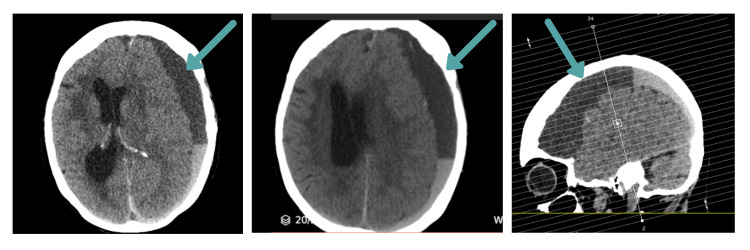
CT head showing rebleeding after COVID-19 infection CT demonstrating worsening acute-on-chronic left subdural haematoma measuring 16 mm with new layering indicating acute rebleeding. Midline shift increased to 12 mm (from 9 mm).

Urgent repeat CT scan demonstrated substantial worsening of the previously identified left subdural haematoma with significant fresh haemorrhage (Figure 3). The haematoma measured 16 mm at maximal thickness with prominent layering visible within the collection. The midline shift had increased to 12 mm. The findings were consistent with acute rebleeding into the chronic subdural collection. Critically, no intervening trauma had occurred between the prior imaging and this acute deterioration.

Given the patient's age, neurological status, and poor prognosis, the family opted for palliative care after extensive discussion. The patient died 10 days after a COVID-19 diagnosis and 17 days after initial presentation. Post-mortem examination was not performed per the family's wishes.

## Discussion

This case represents a rare documentation of spontaneous subdural haematoma rebleeding triggered by COVID-19 infection in the absence of trauma, anticoagulation, or surgical manipulation. The temporal relationship, with catastrophic deterioration occurring within 24 hours of a COVID-19 diagnosis after seven days of clinical stability, raises the possibility that COVID-19 may have contributed to the rebleeding event.

Several features of this case support implicating COVID-19 as a contributing factor. First, the patient demonstrated clinical stability for seven days prior to COVID-19 diagnosis, with plans for rehabilitation discharge. Second, CT imaging performed after the inpatient fall on the second day showed no acute rebleeding despite mechanical trauma, yet CT performed within 24 hours of COVID-19 diagnosis revealed significant acute haemorrhage. Third, the absence of anticoagulation therapy and normal coagulation parameters on admission excludes iatrogenic causes of bleeding.

Melmed et al. in their study of 755 patients with COVID-19 reported the incidence of intracerebral haemorrhage (ICH) in 7.5% patients, mostly associated with anticoagulation [4], while some studies describe COVID-19-associated acute subdural haematomas rather than rebleeding [7-9]. Furthermore, Panciani et al. reported increased rebleeding rates in surgical cases (40%) [10], but spontaneous rebleeding without intervention remains poorly documented.

The catastrophic deterioration observed in our case resulted from several synergistic pathophysiological mechanisms, which are explained further. 

Direct viral endothelial injury

SARS-CoV-2 directly infects cerebral endothelial cells via angiotensin-converting enzyme 2 (ACE2) receptors, causing endotheliitis with cell swelling, disruption, and apoptosis [11]. The subdural haematoma's hyper vascularised outer membrane, containing fragile neo-capillaries formed through pathological angiogenesis [12], provides an especially vulnerable substrate for viral-mediated injury. These immature vessels lack proper basement membranes and pericyte coverage, making them exquisitely sensitive to direct viral damage [13].

Blood-brain barrier disruption

COVID-19's cytokine storm, characterised by IL-6, TNF-α, and IL-1β elevation, damages the neurovascular unit and increases vascular permeability [14]. Studies have shown that matrix metalloproteinase-9 (MMP-9) levels increase significantly in severe COVID-19, with some reports documenting up to 10-fold elevation [15]. MMP-9 degrades vascular basement membranes and type IV collagen, particularly affecting already-compromised vessels in the subdural space [16].

Complement-mediated vascular injury

Classical complement pathway activation causes direct endothelial cell lysis through C5b-9 membrane attack complex deposition. Autopsy studies have demonstrated extensive complement deposition in the cerebral microvasculature of COVID-19 patients, with co-localisation on endothelial cells correlating with microvascular injury and thrombotic complications [17].

Coagulopathy and platelet dysfunction

COVID-19 induces a complex coagulopathy characterised by both thrombotic and haemorrhagic tendencies. The virus directly activates platelets through spike protein interaction, leading to premature degranulation. Additionally, increased von Willebrand factor activity and factor VIII elevation create a prothrombotic state that paradoxically increases bleeding risk [18].

Clinical implications

Patients with pre-existing subdural haematomas who contract COVID-19 may require intensive neurological monitoring, as haemorrhagic complications can occur rapidly (within 24 hours in our case) or may be delayed weeks after apparent viral clearance [19].

Clinicians should consider a low threshold for repeat neuroimaging in COVID-positive patients with known intracranial pathology who develop any neurological change. The management dilemma regarding anticoagulation deserves particular emphasis; COVID-19's prothrombotic state often necessitates therapeutic anticoagulation, yet this dramatically increases intracranial haemorrhage risk in patients with pre-existing subdural collections.

Limitations

This case has several limitations. Causality cannot be established from a single case report, and spontaneous progression of chronic subdural haematoma remains an alternative explanation. Post-COVID coagulation studies and inflammatory markers (CRP, D-dimer, IL-6) were not obtained as the decision for palliative care was made within 24 hours of COVID-19 diagnosis due to catastrophic neurological deterioration. Vascular imaging (CT angiography) was not performed due to the patient's frail status and palliative care pathway, precluding exclusion of other causes such as dural arteriovenous fistula. Despite these limitations, the temporal stability for seven days followed by deterioration within 24 hours of COVID-19 diagnosis, in the absence of intervening trauma, warrants reporting.

## Conclusions

This case documents a temporal association between COVID-19 infection and spontaneous subdural haematoma rebleeding in the absence of trauma or anticoagulation. The rapid deterioration within 24 hours of COVID-19 diagnosis, following seven days of documented clinical stability, raises the possibility that SARS-CoV-2-mediated vascular and inflammatory effects may have contributed to haemorrhagic decompensation. However, causality cannot be established from this single case.

Clinicians should maintain heightened surveillance for haemorrhagic complications in patients with pre-existing subdural haematomas who contract COVID-19. Future prospective studies are needed to determine whether COVID-19 infection increases the risk of haemorrhagic complications in patients with pre-existing intracranial pathology.
